# “*I Must Be the One to Change; He’s Doing the Best He Can*”: Care Partner Evaluation Results from a Four-Part, In-Person, Dementia Community Education Program

**DOI:** 10.3390/ijerph22020295

**Published:** 2025-02-17

**Authors:** Olivia C. Rubio, Erica K. Husser, Rollin Wright, Diane Berish, Janice Whitaker, Marie Boltz, Donna Fick

**Affiliations:** 1 Ross and Carol Nese College of Nursing, The Pennsylvania State University, University Park, PA 16802, USA; deb460@psu.edu (D.B.); jmw5969@psu.edu (J.W.); mpb40@psu.edu (M.B.); dmf21@psu.edu (D.F.); 2 Penn State Health at Hershey Medical Center, Division of Geriatric Medicine, Department of Medicine, Hershey, PA 17033, USA; rwright4@pennstatehealth.psu.edu

**Keywords:** dementia, community-based supports and services, caregiver support, caregiver education

## Abstract

We created a comprehensive, four-part, in-person, interactive community education series to teach informal, unpaid care partners of persons living with dementia (PLWD) how to support their PLWD, negotiate common day-to-day challenges, and navigate predictable situations that arise as the disease progresses over time. The purpose of this qualitative study was to explore the series impact on participant knowledge, care practices, and perceptions of caregiving. Inspired by the U.S. Medicare Cognitive Assessment and Care Plan Service visit and the 4Ms of the Age-Friendly Health Systems Framework, our curriculum focused on (1) expanding knowledge about the disease and disease progression, (2) developing skills to better connect and work with PLWD, (3) self-care for care partners, and (4) sharing resources to support future planning. The program was delivered in three communities in Central Pennsylvania (one rural, one small, and one medium metropolitan) and was attended by 146 individuals. Both session and final qualitative evaluation data were assessed using thematic analysis and five major categories emerged: lessons learned, activating new knowledge, impact and changes, assuming responsibility is challenging, and I need more help. Ongoing education, emotional support, and access to quality assistance for long-term planning are all needed and require sustained support.

## 1. Introduction

Approximately 4% of the noninstitutionalized population over the age of 65 in the United States (U.S.) have a diagnosis of dementia [1]. It is estimated that 7 million people in the U.S. and 282,000 people in Pennsylvania over the age of 65 live with Alzheimer’s disease, the most common type of dementia [2]. Dementia is a syndrome or condition defined as progressive decline in function of at least one cognitive domain (e.g., memory or language) that leads to progressive impairment in a person’s ability to perform higher level skills (e.g., transportation and managing finances) and basic everyday self-care (e.g., bathing or getting dressed) [3]. People living with dementia (PLWD) have a high number of unique care needs given the effect of dementia on the persons’ ability to perform day-to-day activities. An estimated 63% of PLWD receive essential day-to-day direct care and support from informal or untrained, unpaid care partners [4], who provide an average of 31 h of care per week [2]. Care partners also manage other more nuanced aspects of caring for their PLWD, including understanding and managing the person’s changing personality and behavioral symptoms of distress (e.g., anxiety, irritability, delusions), coordinating care with multiple providers, and navigating a future of progressive decline as the PLWD becomes increasingly more dependent [5,6].

Roughly 465,000 informal family care partners of PLWD in Pennsylvania provide 822 million hours of unpaid care per year, valued at a staggering cost of $13.6 billion [2]. Formal care partners, such as nurses, nursing assistants, therapy assistants, and social services professionals, receive months to years of professional training on how to provide day-to-day direct care to patients who cannot meet their own care needs [7]. Informal, family care partners of PLWD are expected to assume these types of roles often with little or no training, experience, or support. It is therefore unsurprising that care partners of PLWD report feeling unprepared to navigate the personality and behavioral symptoms associated with dementia, the demands of direct caregiving, and the complex, changing dynamics of their relationships [8]. If not performed skillfully, attempts to provide care and keep their PLWD safe might trigger potentially harmful emotions and behavioral symptoms of distress, such as yelling or hitting, which not only increases tension within the care partner–PLWD dyad [6], but may contribute to the 1.4 million annual visits by PLWD to emergency departments in the United States [9]. Care partners often turn to providers, such as emergency medical professionals, neurologists, and primary care providers (PCPs), for help in controlling symptoms and easing the burden of dementia, but these providers often rely on pharmacological approaches and use off-label, non-evidence-based psychoactive medications that are associated with increased all-cause mortality and stroke-specific mortality [10]. Most healthcare workforce training programs do not teach non-pharmacological approaches to meeting the unique needs of PLWD [11]. This leaves a gap between what healthcare professionals can share with families and the knowledge and skills families need to best care for their PLWD.

Several educational programs use a variety of modalities (e.g., internet-based, written materials, in-person seminars) designed to fill that gap in care partners’ knowledge and skills [12]. National organizations, such as the Alzheimer’s Association, Alzheimer’s Foundation of America, and Dementia Action Alliance, as well as smaller state and health system level organizations offer intermittent live and more continuously available asynchronous virtual online dementia care training to care partners, sometimes at a cost. One systematic review found that online dementia-based training programs consistently improved care partner knowledge and self-efficacy while reducing care partner burden [13]; however, their effect on competency, skill level, and outcomes are not well understood. Furthermore, access to many of these programs remains an issue, particularly for older care partners, who are caught up in a digital divide, either without access to internet technology, or without digital literacy, or proficiency at accessing internet resources [14,15,16]. Without reliable access to or the ability to use technology, rural care partners in particular are further isolated from training and support resources. Several in-person care partner education programs have been developed and studied, demonstrating efficacy in the support of care partners of PLWD [12]. The goals of all types of education programs are to improve care partners’ ability to navigate dementia-related distress symptoms, to reduce care partner stress, to improve quality of life for both the care partner and the PLWD, and/or to help care partners keep their PLWD at home longer [17,18,19,20,21]. However, challenges exist with access, dissemination, and sustainability. Despite what may seem to be an abundant number of opportunities to learn about dementia care, an estimated 2 out of 3 care partners (66%) reported difficulty in finding resources and support to meet their caregiving needs [2]. This is even more pronounced in rural areas, where care partners report lacking internet or computer access [22,23]. For these reasons, there is ongoing demand for effective in-person training, printed informational resources, and non-digital support services for care partners of PLWD.

We created an in-person, community-based, four-part education series designed to circumvent these issues, and we added a respite opportunity by offering a memory café for PLWD. Our comprehensive program, *Living with Dementia*, was free of charge and supported through the Health Resources and Services Administration’s Geriatric Workforce Enhancement Program (GWEP). Education program development and content was informed by caregiving theories and concepts (e.g., stress and coping, uplifts, and caregiver satisfaction) and the 4Ms of Age-Friendly Health Systems, a geriatric healthcare quality improvement framework [24]. The program included elements of the Positive Approach^®^ to Care, a practice model for dementia caregiving [25], and was structured to address the eight priority elements of Medicare’s Cognitive Assessment and Care Plan Services (CAACPS) visit [26]. Each education session covered one or two elements of the CAACPS visit and was conducted in a mixed format that included didactic lecture, role play, and workshop-like opportunities to develop communication skills, to practice problem-solving, to use caregiving tools, and to engage with other attendees. The series was led by a dementia education specialist, a master’s level academic geriatric nurse, and a geriatrician. We collected session evaluations and a final series evaluation. The purpose of this qualitative study was to explore the series impact on participant knowledge, care practices, and perceptions of caregiving for a PLWD.

## 2. Methods

### 2.1. Program Description

Our four-part in-person community-based educational series was developed for informal care partners of PLWD. *Living with Dementia’s* programmatic aim was to provide comprehensive, essential content through various instructional strategies to promote care partners’ understanding of the disease and their ability to navigate the unique but predictable day-to-day demands of caring for a PLWD. Each session lasted two hours (see Table 1 for session title, purpose, topics, and instructional strategies used). Sessions 1, 2, and 3 were facilitated by 3 presenters: a dementia education specialist, an academic nurse (JW), and a geriatrician (RW). The same 3 presenters facilitated a panel discussion with representatives from an elder law firm, financial planning, Area Agency on Aging, and Alzheimer’s Association during session 4. An optional concurrent Memory Cafe was offered at each session for PLWD so their care partners could attend the education session. Memory Café activities included reminiscence, Opening Minds through Art (OMA), and music and movement; all activities involved intergenerational (IG) engagement, where student volunteers from the Penn State University Nese College of Nursing and College of Medicine received training to pair with and meaningfully engage with a PLWD. The IG activities were led by trained interprofessional facilitators.

### 2.2. Curriculum Development

Curriculum development began with training material developed by the third author (a physician dementia care specialist with five years of experience performing the Medicare Cognitive Assessment and Care Plan Services visit (CAACPS, CPT code 99483) [26] in an internal medicine/geriatrics clinic), in partnership with a dementia education specialist. The material was used to train medical students [27] to work directly with PLWD, and was based on the nine elements of the CAACPS visit (history of cognitive decline, functional history, safety assessment, neuropsychiatric symptom assessment, medication safety assessment, care partner stress, capacity assessment, planning for the future and advance directive, medical complexity), and the Positive Approach^®^ to Care [25]. The MCACPS provided guidance for content and structure, while the Positive Approach^®^ to Care enhanced understanding of the physiological changes that occur with dementia as the biological basis of the functional and behavioral expressions of the disease but focused on leveraging the retained skills of persons living with dementia, emphasizing skill and active engagement rather than loss and passive engagement [25]. Positive Approach^®^ to Care encourages care partners to place the person with dementia and the relationship at the forefront of dementia care [25]. The fifth author, a registered nurse and nationally recognized gerontological educator with leadership expertise in long-term care, joined the two experts above, and the interdisciplinary team reviewed the existing training and adapted content for each session, revising, updating, and adding evidence-based approaches, such as the DICE method [28], and the 4Ms of the Age-Friendly Health System (AFHS): *What Matters*, *Medication*, *Mentation*, and *Mobility* [24]. The final curriculum was shared for review and feedback with the larger team (authors 2, 6 and 7), whose feedback was discussed by interdisciplinary team and integrated into the final materials. Material was presented by all three members of the curriculum team, each one delivering material based on their expertise; all provided both leadership and assistance across instructional strategies.

To increase care partners’ knowledge of age-friendly care and evidence-based approaches that are dementia-friendly, person-centered, and preference-based, each session was introduced with an explanation of the 4Ms, the importance of age-friendly language, and core beliefs related to caring for and living with people with dementia [24,25,29]. These principles were as follows: (1) the relationship between the care partner and the PLWD is the most critical element in any situation, not the outcome of an encounter, (2) being ‘right’ does not necessarily translate into a good outcome, (3) people with dementia are doing the best they can and there is still a lot they can do, (4) people with dementia deserve to maintain identity, exercise autonomy, and live a good life, and (5) care partners are key to making life worth living.

### 2.3. Participant Recruitment and Site Selection

We advertised *Living with Dementia* to informal care partners of PLWD. Attendees learned about the program through various channels, including the Pennsylvania State University website, email announcements, word of mouth, radio and newspaper advertisements, local news media coverage, social media, and flyers that were distributed throughout the communities where the educational series were held. In alignment with the focus of our Geriatric Workforce Enhancement Program (GWEP) to improve care for older adults in rural Pennsylvania, we recruited attendees from three communities in Central Pennsylvania, selected based on geographic size, existing relationships with known agencies serving older adults, and proximity to the university campus and academic medical center where series educators were employed (to minimize program travel costs). One site was in a rural micropolitan area (area includes at least one urban area of at least 10,000 people but less than 50,000 total), with 22.1% of its population aged 65 or older and 82.3% of the total population with broadband internet subscription [30]. The second site was in a small metropolitan area (population less than 250,000), with 16.4% 65 and older, and 85.5% with broadband internet subscription [31]. The third location was in a medium metropolitan area (population 250,000 to 1 million) with 17.7% of its population 65 and older and 89.6% with broadband internet subscription [32]. We offered online registration that could be accomplished independently or by calling a landline for assistance.

### 2.4. Evaluation Data

Paper-based evaluation surveys were administered and collected at the end of each session and a final overall series evaluation was administered after the fourth session to assess overall program impact, including participants’ main takeaways, areas of improvement, and remaining challenges or needs. The surveys were conducted anonymously and in-person. For Sessions 1–3, 14 quantitative and 5 qualitative questions comprised the evaluation surveys; the final evaluation included 31 quantitative and 7 qualitative questions. The qualitative questions were open-ended and required only short answers (i.e., what is one useful thing that you learned that you told someone else?). Appendix A provides a list of the open-ended questions from our session-specific and final evaluations. Sociodemographic data and personal information were not collected, and responses could not be tracked across sessions to assess within-person change. The first author and another research assistant cleaned and transcribed the paper-based evaluation survey data. The data was stored in a secure OneDrive folder. Microsoft Excel was used to assist with data management and analysis. We present only the results from our qualitative analysis in this manuscript.

### 2.5. Qualitative Analysis

A thematic analysis was conducted by the first and second author using all qualitative evaluation data; all authors contributed to the analysis to provide dependability. Thematic analysis does not start with a theoretical framework; instead the themes and categories are derived directly from the data [33]. We describe the analysis process in three main phases: data immersion, theme development, and structured analysis.

#### 2.5.1. Data Immersion

Both researchers independently read through the open-ended responses multiple times to immerse themselves in the data and ensure familiarity [34]. Throughout the initial coding and analysis process, the researchers engaged in reflective journaling, documenting their initial thoughts and impressions with each review [33]. During subsequent reviews, frequently occurring words or phrases were highlighted to capture key concepts [34]. These frequently occurring words or phrases were used to create the initial codes [34].

#### 2.5.2. Theme Development

Once initial codes were complete, we compared and grouped them into meaningful clusters based on their relationship to each other. Themes emerged from studying these meaningful clusters, which were further grouped into categories to organize the findings into a coding scheme and enhance conceptual clarity [34]. The initial draft of the coding scheme consisted of 5 categories and 19 themes. Detailed narrative descriptions were composed to distinguish between the themes. The researchers met frequently to discuss the coding scheme and resolve any discrepancies [33].

#### 2.5.3. Structured Analysis

The initial coding scheme was reviewed by authors 3, 5, 6 and 7 to ensure credibility and dependability [33]. Authors 1 and 2 continued subsequent examination of the categories, themes, and codes for clarity, overlap and discrepancy, with themes conveying similar content or ideas merged into existing themes or categories where appropriate [34]. This process resulted in three iterations of the coding scheme with the final version composed of 5 categories and 15 themes. Strategies such as audit trails, peer debriefing, and reflective journaling were used throughout to ensure trustworthiness of the qualitative findings [33].

## 3. Results

Of the 206 individuals registered, a total of 146 (70.9%) attended one of the three programs. Sixty-four (43.8%) attended the program at a suburban senior center (medium metropolitan area); 51 (34.9%) attended at a suburban continuing care retirement community (small metropolitan area); and 31 (21.2%) attended the program at a rural senior center (micropolitan area). Because this was not formal research, we did not collect demographic data, but we did ask, “Are you a care-partner for a person living with dementia (PLWD)?” Table 2 illustrates the number of evaluations completed and the proportion of those who were self-reported care partners.

**Figure 1:**
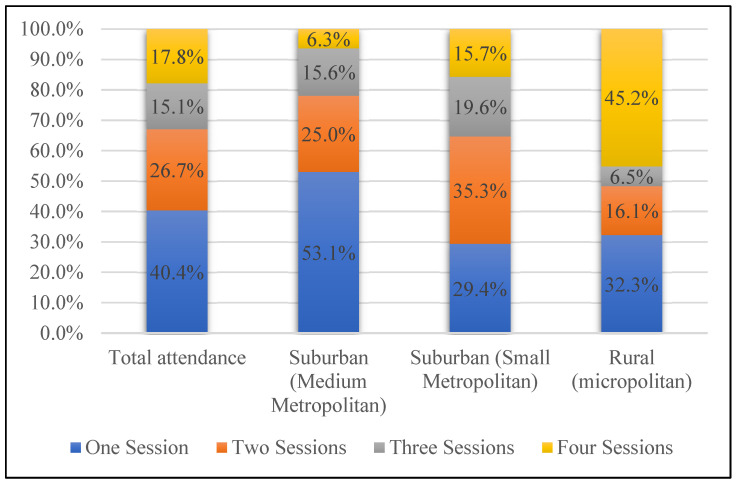
Number of Sessions Attended Per Site Type.

Figure 1 illustrates patterns of attendance across locations. A total of 59 out of 146 (40.4%) participants attended one session across all the locations. A total of 39 out of 146 (26.7%) participants attended two sessions. A total of 22 out of 146 (15.1%) participants attended a total of three sessions. A total of 26 out of 146 (17.8%) participants attended all four sessions. The rural micropolitan senior center had the largest proportion of participants who attended all four sessions (45.2%), compared to the suburban small metropolitan continuing care community where 15.7% attended four sessions, and the suburban medium metropolitan senior center where only 6.3% attended all four sessions. The largest community had the highest proportion of participants who attended just one session (53.1%).

### 3.1. Themes

Thematic analysis revealed five major categories and 15 themes (see Table 3). The five major categories were (1) lessons learned; (2) activating new knowledge; (3) impact and changes; (4) assuming responsibility is challenging; and (5) I need more help. These categories reflect key takeaways, areas of improvement, and remaining challenges and needs following care partners’ participation in the community-based dementia care education series.

### 3.2. Category 1: Lessons Learned

*Lessons learned* reflects participant responses across the first three sessions to the question, “What are two or three of the most important pieces of information you are taking away from this session?” Themes included *Understanding the Disease Process, Simple and Straightforward Communication*, and *Pace and Cues to Enhance Understanding*.

#### 3.2.1. Understanding the Disease Process

Session participants reported improved understanding of the disease progression of dementia, including the physiological brain changes, stages of the disease, and its irreversible nature. They provided specific examples of how the disease impacts cognitive abilities, such as slower processing time, distorted time awareness, diminished language skills, impaired decision-making, and altered visual perception. For example, one participant addressed understanding of the disease progression noting: “*Because of shrinking, realizing decision center function goes first*”. Another participant reported understanding “*how to counter act to better help with the decline by understanding the parts of the brain that are not working*”. Participants reported feeling more knowledgeable about the underlying brain changes that led to behavioral shifts over time in those living with dementia.

#### 3.2.2. Simple and Straightforward Communication 

Participants noted the key communication strategies they had learned to improve interactions with their PLWD. They reported learning to relay their message in a simple and straightforward manner to reduce ambiguity and confusion. They provided examples of communication strategies to lessen the amount of information the brain must process. These included using close-ended questions, minimizing the number of questions, making direct and short statements, and avoiding excessive explanations. For example, one participant stated that: “*I will be more direct and have short easy questions to ask*”. Another reported learning to: “*Think thru conversation—don’t ask open ended questions*”.

#### 3.2.3. Pace and Cues to Enhance Understanding 

Participants also reported learning behavioral strategies to improve the delivery of information to their PLWD. They recognized the importance of slowing down and allowing adequate time for their person living with dementia to process information and reply if appropriate. For example, one participant reported that they learned to, “*Slow down language and wait 10 sec for response*”. Using visual cues alongside verbal communication was shared, in order to reinforce their intended message. A participant reported that they learned to, “*Use more gestures/direction and less words*”. Participants described how they integrated new communication strategies to minimize resistance and anger in their interactions with their PLWD.

### 3.3. Category 2: Activating New Knowledge

Participants described internal shifts that had occurred for them that were impacting how they approached their person living with dementia as *Activating New Knowledge*. Participants shared what they were doing differently as a *Relational Shift from Control to Connection*, and *Emotional Regulation and Reframing*.

#### 3.3.1. Relational Shift from Control to Connection

Participants recognized the importance of valuing their relationship with their PLWD. They described the need to remain flexible when encountering resistance or disagreements. For example, one participant stated, “*Dad is always right! I don’t fight this anymore*”. The participants found that focusing on nurturing a human connection rather than accomplishing the task at hand or their priorities enhanced the relationship. Another participant reported, “*I focused on our relationship more than accomplishing tasks and got better cooperation to actually complete the tasks*”.

#### 3.3.2. Emotional Regulation and Reframing

Participants expressed an understanding that their approach as caregivers impacted the outcome of a given situation. They stressed the need to manage their emotions, practice patience and reduce confrontations. Participants also mentioned strategies such as reframing their questions or statements and redirecting their PLWD when necessary. A participant learned to, “*never point out to him that I’ve already said something five times*”. Another participant reported that they learned, “*how to engage without push back, how to talk without causing anger*”.

### 3.4. Category 3: Impact and Changes

This category illustrates outcomes of activating new knowledge and shifting cognitive schemas. Participants described observing changing dispositions in both themselves and their PLWD, and noted specific improvements made to enhance the environment and quality of life for their PLWD. Themes include *Dyad’s Demeanor and Mood, Environmental Enhancements*, and *Enriching Daily Life*.

#### 3.4.1. Dyad’s Demeanor and Mood 

Participants reported that the integration of new knowledge and caregiving skills, along with their internal shifts, results in more positive outcomes for both themselves and their loved ones with dementia. They reported experiencing increased patience, affection, and thoughtfulness. Participants perceived that they were better equipped to respond respectfully while preserving the dignity of their PLWD. Participants reported observing a noticeable change in the PLWD’s demeanor due to their behavioral adjustments. They noted reduced outbursts, improved cooperation, and enhanced mood. They perceived their loved one with dementia as being friendlier, happier, and calmer. A participant reported: “*She has become friendlier because I’ve learned to touch, hug her more, I learn to connect with her*”. Another participant stated: “*I take things slower so she is more calm*”.

#### 3.4.2. Environmental Enhancements 

Participants described the environmental modifications made to their home to support their PLWD. Some mentioned reducing unnecessary stimulation by organizing clutter and turning off the television. Others discussed employing organizational system and visual reminders, such as calendars, checklists, color-coded systems, and labels, to enhance cognitive support for their loved one with dementia. For example, one participant stated that they: “*Hung a large digital clock with date in dad’s room*”. Another participant stated that they: “*Label objects/drawers*”. Participants also described how they enhanced home safety through structural changes, such as adding handrails, safety gates, shower aids, and improved lighting. For example, another participant stated that they: “*Got brass bar near sink (in bathroom) to grab hold if legs get weak*”. They also reported purchasing walkers and adopted new strategies for medication management.

#### 3.4.3. Enriching Daily Life 

Participants also discussed the behavioral and environmental adjustments they made to bring more activity and joy into the everyday life of their PLWD. This included playing more music and decorating the home to improve quality of life. Participants also reported trying to reduce isolation and providing companionship by scheduling regular social contacts. They carried out activities with their person with dementia such as exercising and crafting. For example, a participant stated that they: “*Help her do word search, she likes doing it together with someone. She loves dogs, recently lost her little dog. I’ve started to occasionally bring my little dog for her*”. Another participant reported that they: “*Join exercise group where we both participate in same session twice a week*”.

### 3.5. Category 4: Assuming Responsibility Is Challenging

Acting as a care partner required them to balance the PLWD’s autonomy with increasing barriers, challenges, or uncertainty in terms of understanding when they needed to take over as they continued to assume primary caregiving responsibilities. Four themes emerged: *Providing Personal Care*, *Ensuring Quality of Life, Making Hard Decisions*, and *Keeping My Person Safe*.

#### 3.5.1. Providing Personal Care 

As the disease advanced and affected functional abilities, the participants noted the difficulty of assuming responsibility for day-to-day physical wellbeing. They described the challenge of helping with personal hygiene, toileting, mobility, diet, sleep, and medications without receiving proper training. In addition to these discrete tasks, one participant stated that their family member, “*needs help getting him moving*”. Another mentioned that they still need help, “*Managing non-dementia medications for other health issues*”, and “*managing care for PWD when you live far away*”. Participants also highlighted the struggle of balancing care with their own personal responsibilities, such as work. One participant stated, “*I can’t care give and take care of myself. I work full-time and try to help mom too. She refuses other help/helpers*”.

#### 3.5.2. Ensuring Quality of Life 

Participants felt it was important to ensure quality of life for their PLWD. They requested more information on appropriate activities to engage in with their person at different stages of the disease. They emphasized the importance of interacting with others and the need to reduce social isolation for their person with dementia. However, they discussed the struggle of finding ways to fill the day with meaningful and stimulating activities. For example, one participant reported that they still needed help with: “*How to get my husband engaged in activities that would stimulate his mind*”. Another participant stated that they needed help: “*Finding more to do (he’s still independent) when I’m at work all day*”.

#### 3.5.3. Making Hard Decisions 

Participants described struggling to make decisions and coping with transitional phases as dementia progresses. They reported grappling with the complexities of knowing when to assume control and make decisions for their person with dementia, while also respecting their autonomy. They shared concerns about navigating difficult conversations surrounding driving, transitioning to a care facility, and long-term planning. For example, one participant asked: “*When do you know to take away car keys?*”. Another participant reported wanting to learn more about: “*Details of how to take over aspects of patient’s daily life (financial accounts, Facebook, email, phone*, etc.*) without interfering with independence and self image*”.

#### 3.5.4. Keeping My Person Safe 

Lastly, participants discussed the challenges that arose when attempting to safeguard their PLWD from external hazards and approaching conversations around risks. They describe difficulties when intervening to address dangerous behaviors, such as approaching issues surrounding alcohol abuse. For example, one participant expressed: “*I’m dealing with cognitive decline brought on by alcohol abuse—how to deal with the alcohol and memory loss*”. Participants also expressed concern over how to protect their person from external threats, such as predatory scammers or telemarketers. For example, one participant reported wanting to learn more about: “*How to talk with person with dementia to convince them that they need assistance. (Mine often falls prey to internet scams and telemarketing and doesn’t understand what an easy mark he has become)*”. Another participant expressed that they wanted to learn more about: “*What legal instruments are available to protect the person with dementia from overly aggressive and self-centered and manipulative/controlling family members*”.

### 3.6. Category 5: I Need More Help

The last major category was in addition to the recognition that participants had gained knowledge and learned new skills. Despite positive experiences and learning success, participants reported needing ongoing teaching and opportunities for skill-building. Themes included *Navigating and Managing the Disease Process, Preparing Others*, and *Finding Available Support*.

#### 3.6.1. Navigating and Managing the Disease Process 

Participants reported ongoing challenges navigating and managing the disease progression. They discussed challenging behaviors that continued to persist as dementia progressed. They expressed wanting more information about how to effectively respond when their person with dementia displayed anger, resistance, paranoia, and inappropriate conduct towards others. For example, one participant wanted to learn more about: “*Paranoid behavior in early stages, how to deal with anger and pinning family against each other*”. Participants also noted ongoing difficulties with trying to understand their person as physiological changes occurred with the disease, impacting functional abilities. They expressed the desire to learn how to more effectively interpret the needs of their person with dementia. They also described the desire to seek a deeper understanding of how their person processes information during social interactions. For example, one participant expressed that they want to learn about: “*Interpreting what person living with dementia’s actual issues are*”. Another participant stated they wanted to learn about: “*Decoding behaviors as language problems increase*”.

#### 3.6.2. Preparing Others 

Participants suggested a need for dissemination of information from the educational series to those who engage with PLWD. This included physicians, staff in personal care and skilled nursing facilities, and school students. They expressed wanting to raise awareness about dementia, foster more positive interactions, and better prepare those who regularly interact with people living with dementia. They also discussed the importance of educating family members and children about how to respond to their loved one living with dementia. For example, one participant stated that it is: “*Extremely valuable for increased community awareness. Should also be increased education for medical providers*”.

#### 3.6.3. Finding Available Support 

Participants recognized the need to plan and expand their care network. However, they expressed that they were struggling with knowing where to find home support, financial, or legal assistance. For example, one participant noted the need for help to: “*Explore ways to set up care team when we are new to community*”. Another participant wanted to learn more about: “*Legal and ethical aspects of dementia care (and financial)*”. They noted the importance of self-care and stressed the need for resources that adequately address caregiver burnout, such as respite care and home support. For example, one participant reported the need for: “*Resources available to help caregivers*”.

## 4. Discussion

We created a community-based, in-person education series, *Living with Dementia*, with the aim of improving care partners’ understanding of the disease and their ability to navigate the unique but predictable day-to-day demands of caring for a PLWD. Our qualitative findings emphasize three primary implications. First, the series helped inform care partners and improve their understanding of the physiological changes that affect brain functions. This knowledge improved participants’ experiences with caregiving and helped them better manage some of the day-to-day challenges by changing their communication techniques, enhancing the environment, and adding enjoyable activities.

Second, the series resulted in a perspective shift for our care partners, who placed emphasis on the importance of the relationship rather than on being right, accomplishing tasks, or trying to control the situation. Care partners reported they had changed their own behavior and, as a result, they reported perceiving a positive change in their loved ones’ demeanor, with less undesirable behavioral symptoms. This finding reflects a key principle of the educational program in alignment with the Positive Approach^®^ to Care. It also reflects generally accepted recommendations to use non-pharmacologic approaches to prevent and manage dementia-related behavioral symptoms rather than off-label use of medications, such as anti-psychotics [35]. Prior literature suggests that positive aspects of caregiving are a distinct part of the caregiving experience that can be fostered through care partner choices and strategies [36]. Positive aspects of caregiving are associated with benefits for the care partner, care recipient, and the care dyad [37]. The approach could be taught to healthcare professionals, who could then better meet the needs of and support families caring for PLWD, thus improving both care partner’s wellbeing and the relationship between the care partner and care recipient [38].

Third, our findings revealed ongoing gaps in dementia care education programs, such as the need for better and more consistent guidance to navigate complex emotions and challenging behavioral symptoms as they arise with disease progression and their relationship with their PLWD changes. Participants frequently voiced the difficulties they faced when stepping into the caregiving role and continuing to provide care for their PLWD with little to no training, experience, or support. This finding is consistent with previous qualitative research, where care partners expressed feeling unprepared to take on the demands of dementia caregiving [8]. Our findings highlight that improved self-efficacy for care partners is just one part of the solution. They also need their communities and health care teams to be more knowledgeable about and supportive of the day-to-day demands of dementia caregiving.

Findings from the Lancet Commission on Dementia Prevention, Intervention, and Care found that carers need education, are not receiving enough education, and are often not aware of available services, especially in under-resourced areas [39]. From our descriptive analyses, we found that almost half (45.2%) of attendees at the rural series attended all four sessions, compared to 8% and 15% at the two suburban locations. Those attending the series at the rural location had less access to technology, which may explain the difference in attendance patterns across sites. This is an important outcome and reinforces the need for in-person education in rural areas, where community members are challenged regarding access to affordable technology and broadband service [22,23]. Online dementia-specific educational and training resources are available for care partners through professional organizations (e.g., Alzheimer’s Association), healthcare systems, and university websites. Yet those living in rural areas are 1.6 times more likely to have limited internet access than their urban counterparts and, even when access is established, digital and health literacy are often barriers to achieving the necessary knowledge gains [40,41]. This intensifies concerns about the equity of online dementia training programs for those in rural or under-resourced areas, where access to reliable internet and technology may be limited. However, in more urban areas, where attendance was less consistent, digital resources may be useful; a person-centered approach to care partner education is needed to ensure equity across settings [24,29].

### 4.1. Practical Implications and Strengths

*Living with Dementia* demonstrated several strengths worth noting. First, it was a community-based education series, open and free to the public in three communities in central Pennsylvania that took place in person, and provided a respite opportunity for care partners that needed support to attend training. Second, it featured mixed teaching methods, such as didactic methods, role playing, and break-out groups, to enhance engagement with the material. Third, the *Living with Dementia* program was comprehensive and covered a broad range of content on many aspects of caring for a PLWD (functional performance, safety concerns, capacity, behavioral symptom assessment, medication management, care partner system support, and future planning).

Other in-person dementia care educational programs have been developed and studied, demonstrating efficacy in the support of care partners of PLWD, improving their ability to navigate dementia-related distress symptoms, reduce burden, and improve quality of life for both the care partner and care recipient [12,17,18,19,20,21,42]; however most programs have delivered information and training in a one-directional approach rather than offering an interactive format that allowed care partners to share their perspectives and needs with the interventionist, thus guiding delivery and implementation of the program [17,19]. Burgio et al. (2003) examined the effectiveness of a skills-based training program using an interactive approach that focused on behavior modification, problem-solving, and cognitive reframing; however, the intervention focused on training the care partner rather than addressing and adapting the needs of the dyad [21]. Bass et al. (2019) evaluated the efficacy of a care consultant coaching program for both care partners and their loved one with dementia and offered guidance on how to navigate complex situations as the disease progressed [20]. Care partners reported feeling less isolated and more confident in their caregiving abilities, and experienced reduced caregiver burden, while persons with dementia reported feeling less embarrassment about their memory problems and increased informal support [20]. However, the intervention was delivered via telephone, email, or regular mail contact. A caregiver training program, similar to our program format, featured an interactive multi-media format, educational manual and implemented brainstorming sessions with care partners in a one day, six-hour training session [42]. Evaluation of the program found that it led to improved knowledge and skills [42].

The positive benefits of these educational programs are not to be undervalued, but our program is evidence that a more robust and comprehensive series, delivered in person, captures the benefits of an in-person learning environment where care partners can work through complex emotions, practice problem-solving among like-minded individuals, and gather real-time feedback from dementia care experts. These aspects are most often lost in online dementia care training.

### 4.2. Limitations

Limitations of an in-person dementia care training program are the logistic and feasibility challenges. First, in-person education and training can only reach a small audience compared to the potential of online education programming. The *Living with Dementia* team planned capacity for up to 60 people at each location; scheduling a time of day, date and venue that works for both the care partners and the *Living with Dementia* team was difficult and program delivery was labor and travel intensive. During this series, the expert team traveled an hour to the rural venue and presented at three locations over two days, including two locations on the same day. Another limitation was the administrative time required to market the series and register participants both online and via telephone. The cost of supporting the academic faculty, whose time was allotted to carry out this education series, and the memory café was another limitation, as this was based on grant funding that expired in 2024. For *Living with Dementia* to be sustainable and accessible to more care partners across the state’s 67 counties, the team would need to streamline logistics further to improve feasibility, and identify a large benefactor, local community benefactors, or other steady funding sources to sustain the program. Finally, the team did not have the long-term capacity to provide ongoing connections and support to these communities, which several care partners requested.

Further research is needed to evaluate *Living with Dementia’s* efficacy and explore its replicability across diverse populations and settings. More work is also needed to develop strategies to improve the program’s scalability and long-term sustainability. The care partners also highlighted areas for improvements in the program evaluation. Future iterations of the program will require additions to the curriculum, such as information and training on (a) how to manage behavioral symptoms when they occur, (b) how to help their PLWD manage their personal care and mobility needs, and (c) how to structure and fill their day with meaningful, purposeful activities that support quality of life for the PLWD. Our findings also point to policy implications, particularly related to expanding community resources and support for care partners and PLWD living in rural, under-resourced areas. Home support, respite care, financial and legal resources, and opportunities for engagement and socialization for PLWD need to be prioritized.

## 5. Conclusions

The *Living with Dementia* program is a key step toward prioritizing non-pharmacological approaches to dementia care that support care partners as they navigate care giving for their PLWD. Care partners were eager and interested in learning information, gaining skills, and identifying resources to help them perform well in their caregiving role. While in-person education for caregivers is a critical ingredient for improving quality care for PLWD, the education needs to be engaging and of high-quality (e.g., mixed teaching methods), accessible, comprehensive (e.g., all topic areas of the Medicare Cognitive Assessment and Care Plan Service), and responsive to the evolving needs of the care dyad, ever-changing community resources, and new technological advances. Overall, we found participants were grateful for the new understanding and tools, but were left wanting more. Ongoing education, emotional support, and access to quality assistance for long-term planning are all needed and require sustained support.

## Figures and Tables

**Table 1:** Overview of the content, learning objectives, and instructional strategies across *Living with Dementia* sessions.

Session Title	Purpose	Topics ^1^	Instructional Strategies
Understanding Dementia-Related Brain Changes	To increase care partners’ knowledge of how the brain changes in dementia	4Ms of Age-Friendly Health Systems Brain function and changes in aging versus dementia (neuropathology) Medical evaluation methods Most common types of dementia Staging and progression of dementia Possible treatment options including state of science on medication use	− Interactive lecture − Role play
2. Understanding Behaviors as a Form of Communication	To recognize that the behaviors of people living with dementia can be interpreted as non-verbal signals of distress, unmet needs, or an attempt to satisfy a need	Stages of dementia with emphasis on retained abilities and care approaches Use of communication and observational skills to better communicate and support PLWD “Behaviors” as signals of distress, unmet needs, or other messages Care approaches to address needs and promote positive well-being Promotion of person-centered and preference-based care approaches Creating a dementia friendly environment at home	− Interactive lecture − Role play − Practicing skills with a partner − Small group problem-solving
3. Resources for Caregivers	To acknowledge and provide strategies and resources to reduce caregiver burden and promote well-being	Causes, signs, and potential impact of caregiver stress Ways to manage emotions, reduce stress, and increase support Building an interdisciplinary dementia care team Respite care and support systems Caregiver safety, risks of abuse, neglect, or dangerous situations Ways to increase safety at home and in the community	− Interactive lecture − Self-assessment tools − Think, pair, share activities − Problem-solving worksheet
4. Planning for the Future with Dementia	To raise care partners’ awareness of the importance of planning for the future, including relevant strategies, information, and resources	Advanced care planning during pre-clinical or early stages of dementia Revisiting and updating plans for care and life during changes in condition and periodically Resources available to assist families with exploring options including: elder law, financial planner, Area Agency on Aging, Alzheimer’s Association, and more Establishing goals related to future planning	− Interactive lecture − Problem-solving worksheet − Panelist discussion

^1^ Material was presented by all three members of the interdisciplinary curriculum team.

**Table 2:** Evaluations Completed by Care Partners.

Session	Total Evaluations Completed	Evaluations Completed by Care Partners *n* (%)
1	72	45 (62.5)
2	70	41 (58.5)
3	52	29 (56.9)
4	57	44 (80)

**Table 3:** Thematic analysis results.

Categories (5 Major)	Themes (15)	Quotes
Lessons Learned	(1) Understanding the Disease Process	“*How to counter act to better help with decline by understanding the parts of the brain*”
	(2) Simple and straightforward communication	“*Use more gestures/direction and less words*”
	(3) Pace and cues to enhance understanding	“*Go slower—use more motions*”
Activating New Knowledge	(1) Relational shift from control to connection	“*Dad is always right! I don’t fight this anymore*.”
	(2) Emotional regulation and reframing	“*How to engage without push back/How to talk without causing anger*”
Impact and Changes	(1) Dyad’s Demeanor and Mood	“*She has become friendlier because I’ve learned to touch, hug her more. I learn to connect with her*”
	(2) Environmental Enhancements	“*Hung a large digital clock with date in dad’s room*”
	(3) Enriching Daily Life	“*Help her do word search, she likes doing it together with someone. She loves dogs, recently lost her little dog. I’ve started to occasionally bring my little dog for her*.”
Assuming Responsibility is Challenging	(1) Providing Personal Care	“*Managing medications—what to take when*”
	(2) Ensuring Quality of Life	“*How to get my husband engaged in activities that would stimulate his mind*”
	(3) Making Hard Decisions	“*When do you know to take away car keys?*”
	(4) Keeping My Person Safe	“*How to talk with a PLWD to convince them that they need assistance. (Mine often falls prey to internet scams and telemarketing and doesn’t understand what an easy mark he has become.)*”
I Need More Help	(1) Navigating and managing the disease progression	“*Decoding behaviors as language problems increase*”
	(2) Preparing others	“*Extremely valuable for increased community awareness. Should also be increased education for medical providers*”
	(3) Finding Available Support	“*Explore ways to set up care team when we are new to a community*”

## Data Availability

The qualitative data presented in this article are not publicly available due to privacy and confidentiality considerations for participants.

## Appendix A. Living with Dementia Evaluation Questions

Session Evaluation Questions (Delivered at sessions 1, 2, and 3)

- In your own words, please provide feedback that will help us improve the program.
- What are two or three of the most important pieces of information you are taking away from this session? (e.g., what information will influence you the most?)
- List two or more things you will do differently because of what you learned today.
- What would you like to learn more about?
- Please share other thoughts or ideas you would like us to hear.

Final Evaluation Questions (Delivered at sessions 4)

- One behavior or characteristic of my PLWD that improved because of what I learned here
- One behavior or characteristic of my PLWD that I still need help with
- Two useful things I learned about how the brain is changing are
- One useful thing I learned that I told to someone else was
- One thing I changed to make our home (or place where my PLWD lives) more dementia-friendly was
- One thing I tried to do to bring structure and meaning to the day for my PLWD was
- Please share other thoughts or ideas you would like us to hear. For example, what topics would you like to learn more about?
